# Circulating Glycan Monosaccharide Composite-Based Biomarker Diagnoses Colorectal Cancer at Early Stages and Predicts Prognosis

**DOI:** 10.3389/fonc.2022.852044

**Published:** 2022-04-29

**Authors:** Haoran Li, Xueling Wang, Xiaodan Huang, Yanli He, Yiran Zhang, Cui Hao, Pengjiao Zeng, Meng Zhang, Yanyun Gao, Dandan Yang, Ming Shan, Huaiqian Dou, Xiaoyu Li, Xiaotian Chang, Zibin Tian, Lijuan Zhang

**Affiliations:** ^1^Department of Hepatobiliary and Pancreatic Surgery, The Affiliated Hospital of Qingdao University, Qingdao, China; ^2^Systems Biology & Medicine Center for Complex Diseases, The Affiliated Hospital of Qingdao University, Qingdao, China; ^3^Department of Gastroenterology, The Affiliated Hospital of Qingdao University, Qingdao, China; ^4^Center for Clinical Research, The Affiliated Hospital of Qingdao University, Qingdao, China; ^5^Shandong Institute of Orthopedics and Traumatology, The Affiliated Hospital of Qingdao University, Qingdao, China

**Keywords:** glycans, monosaccharide composite (MC), colorectal cancer (CRC), diagnosis, prognosis

## Abstract

**Introduction:**

Early diagnosis could lead to a cure of colorectal cancer (CRC). Since CRC is related to aging and lifestyles, we tested if the environmental information-enriched monosaccharide composite (MC) of circulating glycans could serve as an early diagnostic biomarker for CRC. Meanwhile, we evaluated its role in predicting prognosis.

**Methods:**

HPAEC-PAD was used to quantify glycan monosaccharide compositions from a total of 467 serum samples including CRC patients, colorectal adenoma (CRA) patients and healthy individuals. Two diagnostic model was constructed by logistic regression analysis. The diagnostic performance of the two models was verified in the retrospective validation group and the prospective validation group. The prognostic performance of the model was assessed by survival analysis.

**Results:**

The concentrations of monosaccharides in serum were significantly higher in CRA and CRC patients than in healthy individuals. Two diagnostic models were constructed: MC1 was used to distinguish between healthy individuals and CRC; MC2 was used to distinguish between healthy individuals and CRA. Area under receptor operating characteristic curve (AUC) of MC2 and MC1 was 0.8025 and 0.9403 respectively. However, the AUC of CEA between healthy individuals and CRC was 0.7384. Moreover, in early stage of CRC (without lymph node metastasis), the positive rates of CEA and MC1 were 28% and 80%, respectively. The follow-up data showed that the increased MC1 value was associated with poor survival in patients with CRC (*p*=0.0010, HR=5.30).

**Discussion:**

The MC1 model is superior to CEA in the diagnosis of CRC, especially in the early diagnosis. MC1 can be used for predicting prognosis of CRC patients, and elevated MC1 values indicate poor survival.

## Introduction

The mortality and incidence of colorectal cancer (CRC) rank second and third, respectively, among all cancers worldwide (1). And colorectal adenoma (CRA) is considered to be a precancerous lesion. The 5-year survival rate of patients with early CRC after surgical resection is 90% while the advanced CRC was only 10% (2). Thus, early diagnosis is critical to reducing the mortality of CRC.

The current gold standard for diagnosing CRC is colonoscopy combined with pathological biopsy. However, these techniques are challenging to use for early diagnosis due to their invasiveness, requirements for pre-test bowel preparation, and potential complications associated with the procedures.

Glycan structures are composed of monosaccharides in linear or branched sequences with diverse linkages. Since genetic and environmental factors influence glycan structures, they are considered the most information-dense biomolecules for biomarker discovery (3). As both inherited and acquired human diseases are associated with abnormal glycan structures, it was proposed 20 years ago that serum N- and O-linked glycan structures will contribute to the diagnosis and management of cancers and other human diseases (4–6). Interestingly, different *N-*glycan structures in serum IgGs that have no direct relationships with cancer cells can distinguish between healthy individuals and patients suffering different types of cancerous and non-cancerous diseases (7–13). Thus, cancers, including CRC, are systems diseases caused by multiple genetic and environmental factor (14, 15).

Animal glycome is estimated to be 10^4^ times larger than the proteome (16–20). Currently, there are over 800 monosaccharides and 120,000 glycan structures deposited in the GlyTouCan database (21). In theory, serum glycan structures containing systems malfunction information should allow early diagnosis of cancers before cancer cells metastasize into blood circulation. However, glycan structures obtained by the glycomics approach are difficult to translate into clinical use due to the diversity of the glycan structures and other technical and time-consuming issues (22–25).

Abnormal glycan structures have been detected in CRC patients’ sera, tumor cells and tumor tissues (26–29). Moreover, all clinically used circulating cancer biomarkers established during the past 40 years are either specific glycan structures or heavily glycosylated proteins (3, 15). CEA and CA199 are the two most used clinical biomarkers for CRC (30, 31). However, the expression levels of CEA or CA199 in tumors do not correlate with their sera concentrations (32).

There are many different types of serum glycans (3). Indeed, all newly synthesized human glycans consist of ten monosaccharides, which include glucose (Glc), galactose (Gal), glucosamine (GlcN), galactosamine (GalN), fucose (Fuc), mannose (Man), xylose (Xyl), glucuronic acid (GlcA), iduronic acid (IdoA), and 5-N-acetylneuraminic acid or sialic acid (SA). However, few methods have been developed to quantify monosaccharide compositions in human sera as biomarkers. During the past nine years, we have found that the free glucose and mannose concentrations in blood samples (33, 34) reflect glycan homeostasis. We developed various methods to obtain circulating monosaccharide compositions (35–37). We also discovered that monosaccharide compositions can distinguish between cancerous and non-cancerous diseases.

In the current study, the monosaccharide composite (MC) of serum glycans was detected in healthy individuals, CRC and CRA patients. According to the monosaccharide concentration, the diagnostic model for CRC and CRA was constructed. At the same time, we evaluated the diagnostic and prognostic performance of the models.

## Materials And Methods

### Serum Sample Collection

Fasting blood samples were collected. And sera were prepared from both patients and healthy individuals by staff at the clinical lab of our hospital according to doctor’s prescriptions and standardized procedure. The leftover serum samples after clinical tests were collected. Each serum sample was divided into three aliquots and stored at -80°C. Serum was thawed on ice before use.

Moreover, we collected the CEA and CA199 test results from January 2013 to January 2018 from the Clinical Lab of the Affiliated Hospital of Qingdao University for pathologically confirmed CRC patients and healthy individuals. The CEA included 2757 independent test results from healthy individuals and 4,513 independent test results from CRC patients, while CA199 included 930 independent test results from healthy individuals and 4166 independent test results from CRC patients.

The Ethics Committee of Qingdao University has approved our research protocol (QYFYWZLL26178). Informed consent was obtained from both patients and healthy individuals. The study protocol conformed to the ethical guidelines of the 1975 Declaration of Helsinki and was conducted by the guidelines set by the Ethics Committee of the Affiliated Hospital of Qingdao University.

### Study Design

#### Retrospective Cohort

A total of 269 patients were recruited, including 148 cases of CRC and 121 cases of CRA from 2018 to 2019. All patients with CRC or CRA were confirmed by postoperative pathology or pathological biopsy after colonoscopy. We excluded patients who have diabetes, inflammatory bowel disease or any history of treatment. All patients were first diagnosed, and no treatment was received before specimen collection. A total of 145 age- and gender-matched healthy individuals were selected as the control group. Healthy individuals are defined as those who were given a clean bill of health.

In order to construct a retrospective validation group, 90 individuals [healthy individuals (n=23), CRC (n=38), CRA (n=29)] were randomly selected from the above three groups. The remaining patients (CRC=110, CRA =92, healthy individuals =122) were included in the training group. Patients with CRC were classified according to the Eighth AJCC Edition of TNM staging criteria. Stages I and II are defined as early stage, and stages III and IV are defined as advanced stage (38). The information of patients and healthy individuals is shown in **Table 1**.

**Table 1:** Demographic and clinical characteristics of CRA patients, CRC patients, and the healthy individuals.

Characteristics	Mean ± SD or (%)		
	Controls(n=122)	CRA (n=92)	CRC (n=110)
Age, years	60.81 ± 0.70	59.38 ± 0.97	61.93 ± 0.92
Male	78 (63.93%)	61 (66.30%)	72 (65.45%)
CEA, ng/ml	1.21 ± 0.56	2.32 ± 1.43	33.39 ± 13.93
CA199, ng/ml	9.32 ± 2.61	10.78 ± 3.32	43.52 ± 14.92
**CRC location**			
Colon	–	–	51 (46.36%)
Rectum	–	–	59 (53.64%)
**TNM stage**			
I/II	–	–	56 (50.90%)
III/IV	–	–	54 (49.10%)
**CRA pathological type**			
Tubular		53 (57.61%)	
Villous		12 (13.04%)	
Tubulovillous		29 (31.52%)	

CRC, colorectal cancer; CRA, colorectal adenoma.

#### Prospective Cohort

To further evaluate the diagnostic performance of the models established in the above cohorts, 25 CRC patients and 28 healthy individuals were recruited as a prospective validation group. Blinded validation was applied in the prospective group, and other treatments were the same as those applied in the retrospective study cohort.

#### Chemical Reagents

Monosaccharide standards, including Fuc, GalN, GlcN, Gal, Glc, and Man, were purchased from Sigma-Aldrich (USA). HCl (analytical reagent) was purchased from Sinopharm Chemical ReagentCo., Ltd (Shanghai). NaOH solution (50% (w/w)) was purchased from ThermoFisher (Waltham, MA). Deionized water was generated with a Millipore Mingche Q-Gard system (France). HPLC-grade methanol was purchased from EMD Millipore Co. (Billerica, MA).

### Glycan Monosaccharide Composition Analysis of Serum Samples

The method for monosaccharide composition analysis is divided into four parts: (i) serum sample collection, (ii) microwave-assisted acid hydrolysis of serum glycans, (iii) high-performance anion exchange chromatography with pulsed amperometric detection (HPAEC-PAD)-based monosaccharide separation and quantification, and (iv) data analysis. The detailed procedures of serum sample analysis for the current study can be found in Spring Nature Protocol Exchange (35).

### Statistical Analysis

All data were analyzed with SPSS statistical software (version 26.0; SPSS Inc., Chicago, Illinois, USA) and GraphPad Prism 8.3. All quantitative variables in this study were expressed as mean ± standard deviation (SD) unless otherwise stated. The chi-square test tested the qualitative data. Quantitative variables were analyzed using the t-test, Mann-Whitney U test, or the Kruskal-Wallis test. Based on forward stepwise logistic regression analysis, MC-based diagnostic model was identified and constructed. The diagnostic performance of the model was assessed by receiver operating characteristic (ROC) curve analysis. Sensitivity, specificity, and accuracy were calculated using the cut-off values optimally selected based on the ROC curve. The log-rank test was used for survival analysis. Univariate and multivariate Cox proportional hazards models were used to determine prognostic factors. All reported *p* values were two-tailed, and a *p*-value < 0.05 was considered statistically significant.

## Results

### Serum Monosaccharides Differential Expression Between Healthy Individuals vs CRC or CRA Patients

**Figure 1:**
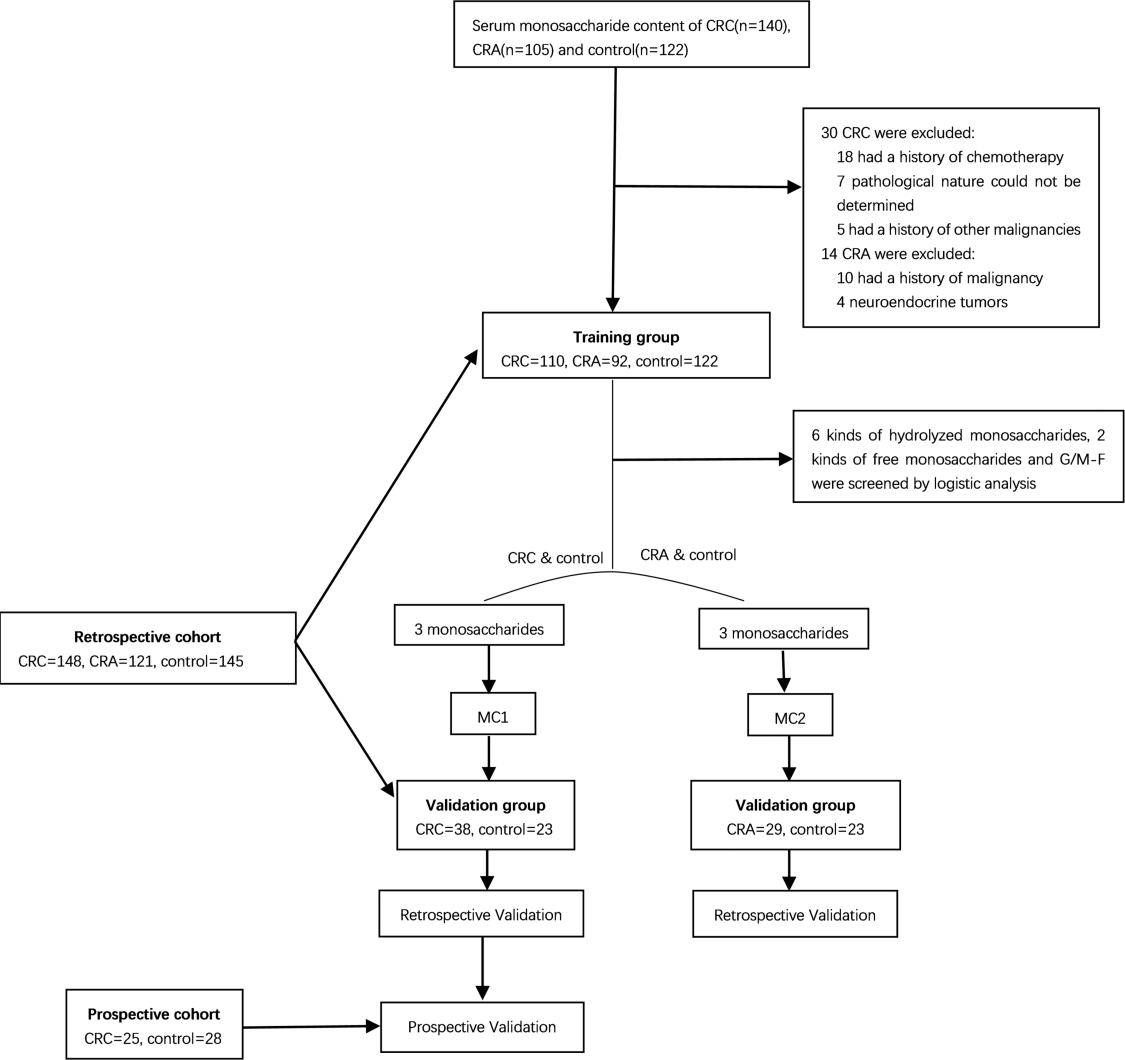
Workflow of the study.

The workflow of the current study for model building and validation are shown in **Figure 1**.

**Figure 2:**
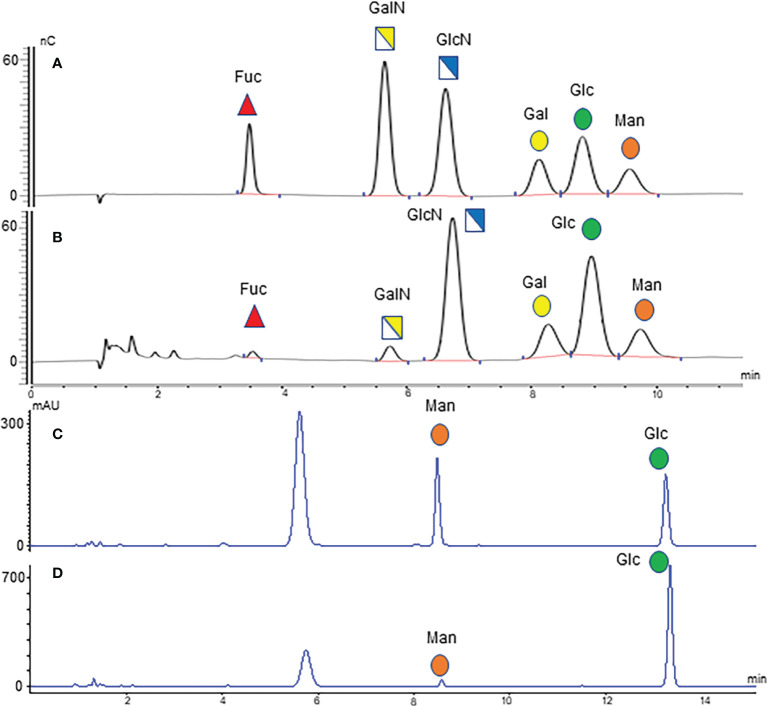
Monosaccharide chromatograms. **(A)** HPAEC-PAD chromatogram of monosaccharide standard; **(B)** HPAEC-PAD chromatogram of serum hydrolyzed monosaccharides; **(C)** HPLC chromatogram of monosaccharide standard; **(D)** HPLC chromatogram of serum free monosaccharides.

**Figure 3:**
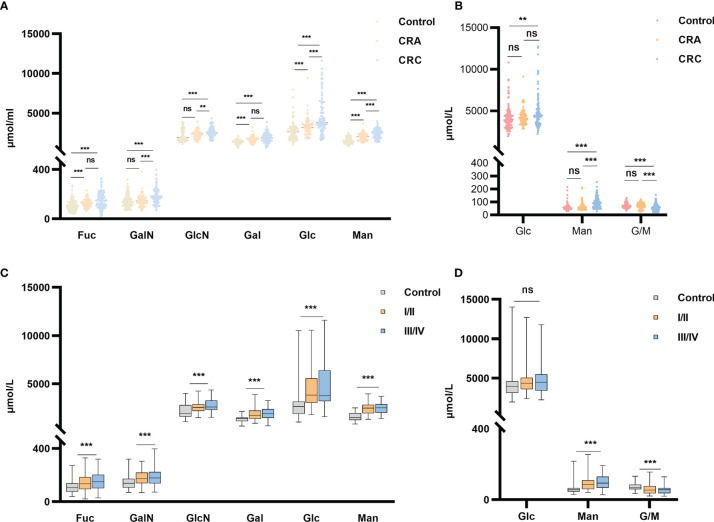
Free and hydrolyzed glycan monosaccharide concentrations in sera of healthy individuals, CRC and CRA patients. **(A)** Six hydrolyzed monosaccharides in sera of the healthy individuals, CRA and CRC groups. **(B)** Two free monosaccharides and their ratio in sera of the healthy individuals, CRA and CRC groups. **(C)** Six hydrolyzed and **(D)** Two free monosaccharide concentrations in sera of healthy individuals and CRC at stages I/II vs. stages III/IV. ***p ≤ 0. 001, **p ≤ 0.01, ns, not significant.

The optimized MAAH plus HPAEC-PAD method (35) was used to quantify the concentrations of two free monosaccharides (Glc-F and Man-F) and the six hydrolyzed monosaccharides (Fuc-H, GalN-H, GlcN-H, Gal-H, Glc-H, and Man-H) in the sera of healthy individuals, CRA, and CRC patients. Chromatograms of free and hydrolyzed monosaccharides are shown in **Figure 2**. Significantly increased concentrations of Fuc-H, GalN-H, GlcN-H, Gal-H, Glc-H, and Man-H in serum were observed in CRC patients compared with healthy individuals. In addition, concentrations of Fuc-H, Gal-H, Glc-H, and Man-H in serum were significantly higher in CRA patients than healthy individuals (**Figure 3A**). Significantly increased Glc-F and Man-F concentration and decreased G/M ratio were observed in CRC groups compared with healthy individuals. Significantly increased Man-F concentration and decreased G/M ratio also presented in CRA groups compared with heathy individuals (**Figure 3B**).

In addition, concentrations of all monosaccharides except Glc-F were higher in early or advanced CRC than in healthy individuals (**Figure 3C**). While G/M ratio was lower in early and advanced CRC than in healthy individuals (**Figure 3D**). However, there is no significant difference in serum monosaccharides between colon and rectal cancer patients. (**Supplemental Figure 1****).**

### Serum Monosaccharides as Diagnostic Biomarkers

#### Serum Monosaccharides Have Better Diagnostic Performance for CRC Than CEA

**Figure 4:**
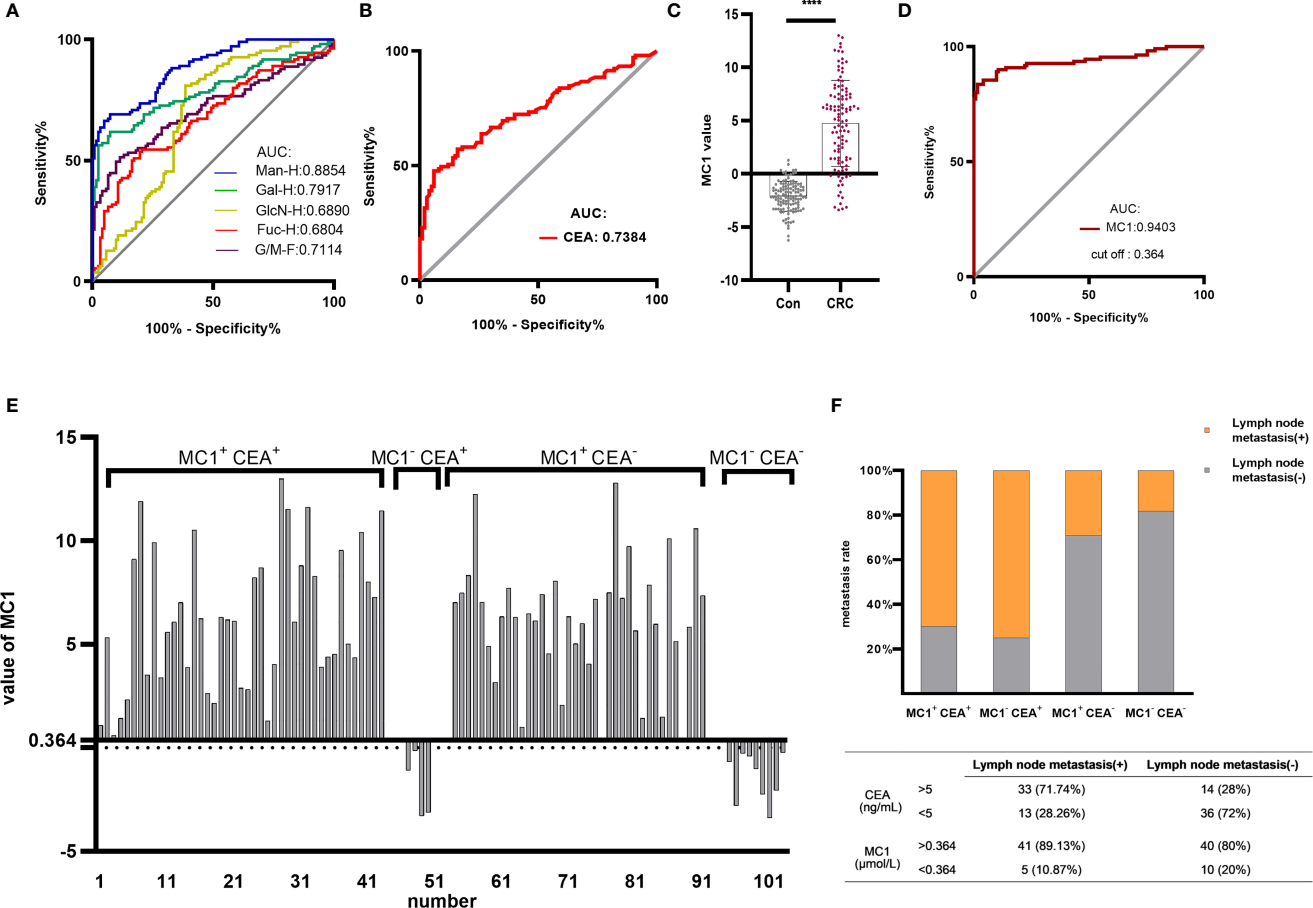
Establishing the MC1 diagnostic model for CRC and evaluating its diagnostic performance. ROC analysis differentiated healthy individuals from CRC patients using monosaccharides **(A)** and CEA **(B)**. Based on the serum monosaccharides concentrations in healthy individuals and CRC patients, a diagnostic model named MC1 was constructed by logistic regression analysis. **(C)** MC1 values were compared between healthy individuals and CRC patients in the training group. **(D)** ROC curve analysis of MC1 was performed to differentiate healthy individuals from CRC patients. The cut-off value (0.364) of MC1 in ROC curve analysis was used as the critical value. MC1>0.364 was defined as MC1^+^, otherwise, it was MC1^-^; meanwhile, CEA>5ng/mL was defined as CEA^+^, otherwise, it was defined as CEA^-^. **(E)** Patients in the training group were divided into four groups: MC1^+^ CEA^+^, MC1^-^ CEA^+^, MC1^+^ CEA^-^ and MC1^-^ CEA^-^, and **(F)** the lymph node metastasis rate in each group was counted. ****p ≤ 0.0001.

Based on the above results, ROC curve analysis was performed to distinguish between healthy individuals and CRC patients using different monosaccharide. The areas under ROC curve (AUC) of Man-H, Gal-H, GlcN-H, Fuc-H and G/M ratios were 0.8854, 0.7917, 0.7114,0.6890 and 0.6804, respectively (**Figure 4A**). The AUC values of Man-H and Gal-H were higher than CEA (AUC=0.7384) (**Figure 4B**). The results suggest that serum monosaccharides may be better diagnostic biomarker for CRC compared with CEA.

To test if the low AUC value shown in **Figure 4B** for CEA was due to the specific cohort used in the current study, we performed the following validation. CEA and CA199 test results of pathologically confirmed CRC patients and healthy individuals were collected from the Clinical Lab of the Affiliated Hospital of Qingdao University. The CEA data included 2757 independent test results from healthy individuals and 4,513 independent test results from CRC patients. The CA199 data included 930 independent test results from healthy individuals and 4166 independent test results from CRC patients. **Supplemental Figure 2** showed that the AUCs were 0.7059 and 0.6176 for CEA and CA199, respectively. The result of CEA was in line with the AUC shown in **Figure 4B**.

#### Establishment of MC1 Diagnostic Model and Evaluation of Its Diagnostic Performance

The above results suggest that MC is a potential CRC biomarker. In the training group, logistic regression analysis was conducted to establish MC-based diagnostic model for CRC (MC1). The logistic regression equation for MC1 was as follows: MC1 = -8.18 - 0.021 Fuc-H - 0.004 GlcN-H + 0.011 Man-H, where the Fuc-H, GlcN-H and Man-H were in μmol/L. **Figure 4C** shows that the MC1 value of CRC patients was significantly higher than healthy individuals. ROC curve analysis was performed to differentiate CRC patients from healthy individuals using MC1 value in the training group (**Figure 4D****)**. The results showed that the AUC of the MC1 was 0.9403, which was higher than CEA.

ROC curve analysis of MC1 in the training group showed that the cut-off value was 0.364. And it was used as the critical value for the CRC diagnosis. MC1>0.364 was called MC1 positive (MC1^+^), MC1<0.364 was called MC1 negative (MC1^-^). MC1^+^ patients were diagnosed with CRC, and MC1^-^ patients were diagnosed with CRC-free. Based on the above definitions, the sensitivity, specificity and accuracy of MC1 in the training group were calculated to be 83.64%, 98.36% and 91.34%, respectively (**Table 2**). Subsequently, the sensitivity, specificity and accuracy of MC1 in the retrospective and prospective validation groups were calculated to be 86.84%, 100%, 91.8%; and 64%, 96.43%, 81.13%, respectively (**Table 3**).

**Table 2:** Diagnostic power of MC1 and MC2 in the training groups.

MC	AUC	Cut-off	*Se (%)*	*Sp (%)*	*PPV (%)*	*NPV (%)*	*Accuracy (%)*	*p* value
MC1	0.9403	0.364	83.64	98.36	97.87	86.95	91.34	<0.0001
MC2	0.8025	1.282	56.04	95.08	89.47	74.35	78.04	<0.0001

MC1, diagnostic model for distinguishing CRC from healthy individuals; MC2, diagnostic model for distinguishing CRA from healthy individuals; CRC, colorectal cancer; AUC, area under the receiver operating characteristic curve; Se, sensitivity; Sp, specificity; PPV, positive predictive values; NPV, negative predictive values.

**Table 3:** Diagnostic power of MC1 and MC2 in the retrospective validation and(or) prospective validation groups.

	Cohort	Test	Actual status		*Se (%)*	*Sp (%)*	*PPV (%)*	*NPV (%)*	*Accuracy (%)*
			(+)	(–)					
MC1	Retrospective	CRC (+)	33	0	86.84	100.00	100.00	82.14	91.80
		CRC (-)	5	23					
	Prospective	CRC (+)	16	1	64.00	96.43	94.12	75.00	81.13
		CRC (-)	9	27					
MC2	Retrospective	CRA (+)	16	2	55.17	91.30	88.89	61.76	71.15
		CRA (-)	13	21					

MC1, diagnostic model for distinguishing CRC from healthy individuals; MC2, diagnostic model for distinguishing CRA from healthy individuals; CRC, colorectal cancer; CRA, colorectal adenoma; Se, sensitivity; Sp, specificity; PPV, positive predictive values; NPV, negative predictive values.

According to the availability of data, 94 preoperative CEA test reports were obtained from CRC patients in the training group. The clinically applied CEA critical value is 5ng/mL. We define CEA>5ng/mL as CEA positive (CEA^+^), CEA<5ng/mL as CEA negative (CEA^-^). MC1^+^ and MC1^-^ are defined as described earlier. We classified the 94 CRC patients into four groups: MC1^+^ CEA^+^, MC1^-^ CEA^+^, MC1^+^ CEA^-^ and MC1^-^ CEA^-^. The specific grouping is shown in **Figure 4E**. Among the 47 CEA^+^ patients, 43 MC1 values were also positive. In addition, 38 of the 47 CEA^-^ patients had positive MC1 values. **Figure 4F** shows the lymph node metastasis rate of patients in the four groups. In the advanced stage of CRC (with lymph node metastasis), the positive rates of CEA and MC1 were 71.4% and 89.1%, respectively. And in the early stage of CRC (without lymph node metastasis), the positive rates of CEA and MC1 were 28% and 80%, respectively. This result indicates that the diagnostic efficiency of MC1 is higher than that of CEA, especially in the diagnosis of early CRC.

#### The Diagnostic Effect of Serum Monosaccharides on CRA and the Establishment of MC2 Diagnostic Model

**Figure 5:**
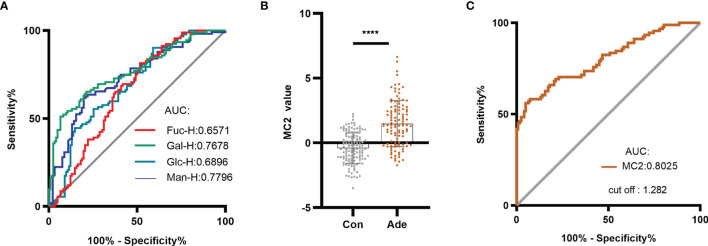
Establishing the MC2 diagnostic model for CRA and evaluating its diagnostic performance. **(A)** ROC analysis distinguished between healthy individuals and CRA patients using serum monosaccharides. Based on the serum monosaccharides concentrations in healthy individuals and CRA patients, a diagnostic model named MC2 was established by logistic regression analysis. **(B)** MC2 values were compared between healthy individuals and patients with CRA in the training group. **(C)** ROC curve analysis of MC2 was performed to differentiate healthy individuals from CRA patients. ****p ≤ 0.0001.

Some of the serum monosaccharides concentrations in CRA were higher than healthy individuals. The ROC curve analysis was performed to differentiate CRA patients from healthy individuals using different monosaccharide. The AUC values of Man-H, Gal-H, Glc-H, and Fuc-H were 0.7796, 0.7678, 0.6896 and 0.6571, respectively (**Figure 5A**). This result indicates that serum monosaccharides may have a diagnostic effect on CRA.

Based on the above result, a diagnostic model of CRA in the training group was constructed by logistic regression analysis and named it MC2. The logistic regression equation for MC2 was as follows: MC2= -6.639 - 0.022 Fuc-H + 0.003 Gal-H + 0.003 Man-H, where the Fuc-H, Gal-H and Man-H were in μmol/L. **Figure 5B** shows that the MC2 value of CRA patients was significantly higher than healthy individuals. In the training group, ROC curve analysis was performed to distinguish healthy individuals and CRA patients using MC2 (**Figure 5C****)**. The result showed the AUC was 0.8025, and the cut-off value was 1.282. The sensitivity, specificity and accuracy of MC2 were 56.04%, 95.08% and 78.04%, respectively (**Table 2**). Subsequently, the diagnostic performance of MC2 was verified in a retrospective validation group, and the sensitivity, specificity and accuracy were 55.17%, 91.30%, and 71.15%, respectively (**Table 3**). It indicates that MC2 can be used as a diagnostic model to identify some CRA patients.

### The Prognostic Role of MC1 in CRC Patients

**Figure 6:**
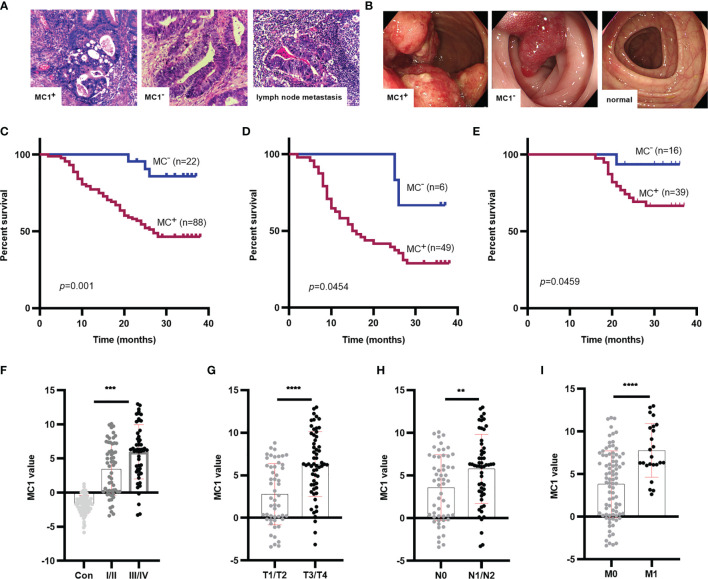
Survival analysis for MC1 values in CRC patients, and MC1 values in different T, N, M stage of CRC. **(A)** The typical pathological data for patients who were MC1^+^, MC1^-^, or suffering lymph node metastasis. **(B)** The colonoscopy data from patients who were MC1^+^, MC1^-^, or colonoscopy from healthy individuals. **(C)** Log-rank survival analysis showed that MC1^+^ was associated with poor prognosis in CRC patients. To determine whether the role of MC1 in predicting survival in patients with CRC is related to tumor progression, the prognostic role of MC1 in early CRC (I/II stage) and advanced CRC (III/IV stage) was evaluated. **(D, E)**: MC1^+^ is associated with poor prognosis of CRC in both early and advanced stage. **(F)** Serum MC1 values in healthy individuals and CRC patients at stage I/II or III/IV. Serum MC1 values in different **(G)** T stage (T1/T2: the tumor did not penetrate the muscularis propria; T3/T4: the tumor penetrated the muscularis propria to the subserosal, visceral layer of peritoneum or directly invaded or adhered to other organs or structures), **(H)** N stage (N0: no lymph node metastasis; N1/N2: at least one lymph node metastasis), **(I)** M stage (M0: no distant organ metastasis; M1: with distant organ metastasis). ****p ≤ 0.0001, ***p ≤ 0.001, **p ≤ 0.01.

We followed up CRC patients in two cohorts and obtained follow-up data of 110 patients. The clinical pathological data was collected by searching pathology reports, pathological sections (**Figure 6A**) and colonoscopy data (**Figure 6B**). At the same time, we learned about the survival status of patients through telephone follow-up and hospitalization records inquiries. Survival analysis showed that MC1^+^ was significantly associated with the poor prognosis of CRC patients (*p*=0.0010, HR=5.30) (**Figure 6C**). Subsequently, to determine whether the effect of MC1 on patient survival was affected by the TNM stages, the prognostic potential of MC1 was separately evaluated in early stage (stages I/II) and advanced stage (stages III/IV) patients. Interestingly, whether in the advanced stage (III/IV) or early stage (I/II), MC1^+^ patients have a lower survival rate (*p*=0.0454, HR=2.45; *p*=0.0459, HR=3.18, respectively). (**Figures 6D, E**).

Furthermore, we analyzed the relationship between MC1 value and clinicopathological indicators. We collected clinicopathological indicators such as sex, age, T stage(T1/T2: the tumor did not penetrate the muscularis propria; T3/T4: the tumor penetrated the muscularis propria to the subserosal, visceral layer of peritoneum or directly invaded or adhered to other organs or structures), N stage(N0: no lymph node metastasis; N1/N2: at least one lymph node metastasis), M stage(M0: no distant organ metastasis; M1: with distant organ metastasis), TNM stage, CEA and albumin-bilirubin (ALBI) grade. ALBI grade is an index to assess liver function based on serum total bilirubin and albumin levels. It was proposed in 2015, and its prognostic value was validated in multiple large international cohorts (39). The ALBI grade is divided into three levels. The higher the ALBI grade, the worse the liver function (39, 40). After analyzing the relationship between MC1 value and clinicopathological indicators, we found that MC1 value was related to TNM stage (*p*=0.0010), T stage (*p*<0.0001), N stage (*p*=0.0044) and M stage (*p*<0.0001) (**Figures 6F–I**; **Table 4**). Univariate COX proportional hazards analysis (**Table 5**) revealed that MC1(HR 5.30; 95% CI 2.83 to 9.95; *p*=0.0010), T stage (HR 3.01; 95% CI 1.76 to 5.16; *p*=0.0002), N stage(HR 4.45; 95% CI 2.56 to 7.73; *p*<0.0001), M stage(HR 11.24; 95% CI 3.87 to 32.63; *p*<0.0001) and CEA(HR 2.28; 95% CI 1.32 to 3.95; *p*=0.0022) were associated with poor prognosis. In multivariate analysis that included MC1, T stage, N stage, M stage and CEA, MC1^+^(HR 3.38; 95% CI 1.02 to 11.22; *p*=0.046) was significantly associated with poor survival and was independent of other clinical factors.

**Table 4:** Clinical significance of MC1 in patients with CRC.

	MC1			
	n	Mean± SD	95%CI	*p* Value
Sex				
Male	72	4.20 ± 4.11	3.23 to 5.17	0.0903
Female	38	5.59 ± 3.97	4.29 to 6.90	
Age(years)				
≤Median (63)	58	4.48 ± 4.15	3.39 to 5.57	0.5900
>Median (63)	52	4.90 ± 4.06	3.77 to 6.03	
T Stage				
T1/T2	50	2.89 ± 3.54	1.88 to 3.89	<0.0001
T3/T4	60	6.17 ± 3.95	5.15 to 7.19	
N Stage				
N0	55	3.58 ± 3.87	2.54 to 4.63	0.0044
N1/N2	55	5.78 ± 4.06	4.68 to 6.88	
M Stage				
M0	86	3.81 ± 3.92	2.97 to 4.66	<0.0001
M1	24	7.78 ± 3.13	6.46 to 9.10	
TNM Stage				
I/II	55	3.46 ± 3.85	2.44 to 4.48	0.0010
III/IV	55	5.99 ± 3.98	4.90 to 7.09	
CEA (ng/mL)				
<5	58	4.25 ± 4.33	3.11 to 5.39	0.2084
>5	52	5.23 ± 3.73	4.19 to 6.27	
ALBI grade				
1	58	4.29 ± 4.25	3.17 to 5.40	0.2200
2	52	5.24 ± 3.78	4.19 to 6.29	

ALBI grade, albumin-bilirubin grade.

**Table 5:** Association between MC1 and prognosis in patients with CRC.

	Univariate			Multivariate		
	HR	95%CI	*p* value	HR	95%CI	*p* value
Age (>median vs <median)	1.51	0.88 to 2.59	0.1038			
Sex (male vs female)	0.92	0.52 to 1.63	0.7724			
T stage (T3/T4 vs T1/T2)	3.01	1.76 to 5.16	0.0002	0.99	0.49 to 2.00	0.9690
N Stage (N1/N2 vs N0)	4.45	2.56 to 7.73	<0.0001	3.27	1.65 to 6.49	0.0010
M Stage (M1 vs M0)	11.24	3.87 to 32.63	<0.0001	21.76	9.34 to 50.71	<0.0010
CEA (>5 vs<5)	2.28	1.32 to 3.95	0.0022	1.82	1.01 to 3.28	0.0480
ALBI grade (2 vs 1)	1.40	0.81 to 2.41	0.2146			
MC1(<0.364 vs >0.364)	5.30	2.83 to 9.95	0.0010	3.38	1.02 to 11.22	0.0460

ALBI grade, albumin-bilirubin grade.

## Discussion

Glycan structures contain both systems and tumor information (41), making them the most attractive candidates for serum cancer biomarker discovery. In contrast, current glycomics approaches depend on profiling complicated glycan structures. However, it is not suitable for clinical application because of cumbersome and non-standard procedure. In addition, the separation and characterization of glycan isomers and the integration analysis of complex samples in high-throughput setting remain challenging (42, 43).

The “OMICS” technologies, including genomics (44), epigenomics (45), proteomics (46), glycomics (47) and metabolomics (48), use the big data approach (49) for serum biomarker discovery. However, few new cancer biomarkers have been introduced into clinical practice during the last 20 years (50). Lemos et al. pointed out that the current methods of screening biomarkers through big data often have “false discovery” and “omission” due to the lack of strategies for large-scale biomarker discovery and validation. It’s like “running before we walk”. The alternative approach focuses on disease-related molecules and implements marker screening through mature detection techniques. It is called “walking before we run” (49). Our quantitative and straightforward assay followed by regression analysis represented such an approach.

In the current study, we used a simple and quantitative assay to obtain glycan monosaccharide compositions of serum samples. Subsequently, MC were used to establish a diagnostic model of CRC (MC1) through stepwise logistic regression analysis. The AUC of MC1 in the training group was 0.9403, which was significantly higher than CEA (0.7384). The accuracy of MC1 in the training group, retrospective validation group and prospective validation group was 91.34%, 91.8% and 81.13%, respectively. It proves that MC1 has good diagnostic performance for CRC.

CEA is the most commonly used marker of CRC in clinical practice. However, CEA is mainly elevated in advanced CRC, which is not conducive to the early diagnosis of the disease (51). In our research, the positive rate of MC1 in early CRC patients (without lymph node metastasis) was significantly higher than that of CEA (80% vs 28%), indicating that MC1 was reliable for CRC early detection. Further, the MC1 could predict the prognosis of CRC patients. The survival rate of MC1^+^ patients was significantly lower than that of MC1^-^ patients (*p*=0.0010, HR=5.30). The COX proportional hazards analysis showed that MC1 was an independent risk factor for poor survival in CRC.

In addition, we constructed a diagnostic model of CRA and named it MC2. The sensitivity and specificity of MC2 in the training group and retrospective validation group were 56.04%, 95.08%; 55.17%, 91.3%, respectively. It indicates that MC2 has a particular diagnostic value on CRA as well. We demonstrated the ability of a MC-based diagnostic model to diagnose benign colorectal tumors. Whether this diagnostic model can be used as a clinical application requires rigorous evaluation.

Serum glycans in the form of glycoproteins are mainly synthesized in the liver. However, in our study, there was no significant relationship between MC1 and the liver functional status of patients. As a glycosylated protein, CEA also has no significant relationship with MC1. Indeed, the immunoglobulins such as IgGs that are not synthesized in the liver also contribute a significant amount of glycans in blood circulation (3). And the ALBI grades of our patients were mainly graded I and II, indicating no or slight liver function damage (39). The damage of liver function will affect the glycan structure of glycoprotein (3), but in our study, no significant relationship between MC1 and the liver functional status of patients was found.

In addition, all tissue and organs contribute to the glycans in the blood circulation (52). During the past, the changes of glycosylated genes in cancer cells and tumor tissues have been mainly used to explain the changes in serum glycan structure (29). However, such opinion ignores the non-template synthesis nature of glycan. Glycan structure is not only affected by genes, but also largely affected by nutrition, microenvironment, etc. (3). Indeed, the development and progression of CRC are a multi-step process of hyperplasia, benign tumors, carcinoma in situ, and invasive carcinoma during an extended period (53). It demonstrates that the development of cancer is a lengthy process and cancer is the end-stage of the process. Current cancer markers, such as CEA, only change when the cancer appears or even in the advanced stages. Our results showed that the concentrations of monosaccharides change in both CRA and CRC patients. And the MC1 served as a better biomarker for CRC than CEA, supporting the notion that cancers should be considered the end-stage of systems diseases. The systems malfunction information content in the monosaccharide composite explained why it could serve as a biomarker for early detection of CRC in our current study.

Indeed, sialic acid, a cancer biomarker used in clinical practice, is also a monosaccharide (54). But no new monosaccharide markers have been discovered since then. The bottleneck of monosaccharide composition analysis is to optimize the serum glycan hydrolysis conditions. The microwave-assistant acidolysis assay used in the current study takes 10 min and 10 μl of acid and 2 μl serum to complete the reaction (35). However, the four monosaccharides, SA, Xyl, GlcA, and IdoA, were below the detecting limits for certain serum samples. Thus, these monosaccharides were not used for data analysis in the current study. Despite the limitations, we found that the two free and six hydrolyzed monosaccharides were sufficient to allow us to use the MC1 as a biomarker for CRC.

In the prospective validation group, the sensitivity and accuracy of MC1 decreased compared to the training group. Combined with literature reports and our experimental process, it may be related to the small sample sizes in the current study (49). The result shows that it is essential to establish prospective validation for marker screening, which is also consistent with that advocated by Qian J, et al. (38). Although the Affiliated Hospital of Qingdao University is a regional medical center, it is still possible that patients collected from the same hospital have similar dietary habits, genetic backgrounds, etc. Subsequent multi-center validation with a large sample size can be carried out to further clarify the diagnostic performance of the MC1 model, thereby providing sufficient theoretical support for its clinical application.

In summary, the concentrations of serum monosaccharides in patients with CRC and CRA were higher than in healthy individuals. We constructed a monosaccharide diagnostic model MC1 for the diagnosis of CRC. The diagnostic performance of MC1 for CRC is better than CEA, especially in early diagnosis. In addition, Cox proportional hazards analysis suggests that MC1 is an independent risk factor for the poor prognosis of CRC. Meanwhile, we constructed a monosaccharide diagnostic model MC2, which has a particular diagnostic effect on CRA. In this study, novel diagnostic and prognostic markers of CRC have been identified by serum monosaccharide detection. It suggests that in the future, the reliable and straightforward monosaccharide quantification method is anticipated to be used for more biomarker discoveries for other types of cancers.

## Data Availability Statement

The original contributions presented in the study are included in the article/**Supplementary Material**. Further inquiries can be directed to the corresponding authors.

## Ethics Statement

The studies involving human participants were reviewed and approved by Ethics Committee of The Affiliated Hospital of Qingdao University. The patients/participants provided their written informed consent to participate in this study.

## Author Contributions

LZ designed, supervised the study, and wrote the paper. ZT and XC designed and supervised the study. HL and XW participated in the research design, conducted experiments, and drafted the paper. XH, YH, and YZ performed the experiments. CH, PZ, and MZ are involved in the statistical analysis. YG, MS, DY, HD, and XL collected pathological specimens. XL involved in clinical data collection. All authors have approved the final draft submitted.

## Funding

This research was supported by the Natural Science Foundation of China (Grants 81672585), the Qingdao Postdoctoral Application Research Project (Grant No. RZ1900013688), and the Youth Research Fund Affiliated Hospital of Qingdao University, and the Taishan Scholar Fellowship to LZ.

## Conflict of Interest

The authors declare that the research was conducted in the absence of any commercial or financial relationships that could be construed as a potential conflict of interest.

## Publisher’s Note

All claims expressed in this article are solely those of the authors and do not necessarily represent those of their affiliated organizations, or those of the publisher, the editors and the reviewers. Any product that may be evaluated in this article, or claim that may be made by its manufacturer, is not guaranteed or endorsed by the publisher.

## Supplementary Material

Supplementary Figure 1 Monosaccharide concentrations in sera of colon cancer and rectum cancer had no significant difference. The monosaccharides labeled “- F” are free monosaccharides; Monosaccharides without “- F” labeling are hydrolyzed monosaccharides. ****p ≤* 0. 001, ***p* ≤ 0.01, **p* ≤ 0.05, ns, not significant.

Supplementary Figure 2 ROC curve analysis of CEA **(A)** and CA199 **(B)** based on data from January 2013 to January 2018. **(A)** ROC curve analysis based on clinical CEA data of 2757 healthy individuals and 4513 CRC patients. **(B)** ROC curve analysis based on clinical CA199 data of 930 healthy individuals and 4166 CRC patients.
