# Supplementary figures and images for: Circulating Glycan Monosaccharide Composite-Based Biomarker Diagnoses Colorectal Cancer at Early Stages and Predicts Prognosis
Source: Front Oncol. 2022 Apr 29;12:852044. doi: 10.3389/fonc.2022.852044 (PMC9099097; doi:10.3389/fonc.2022.852044)

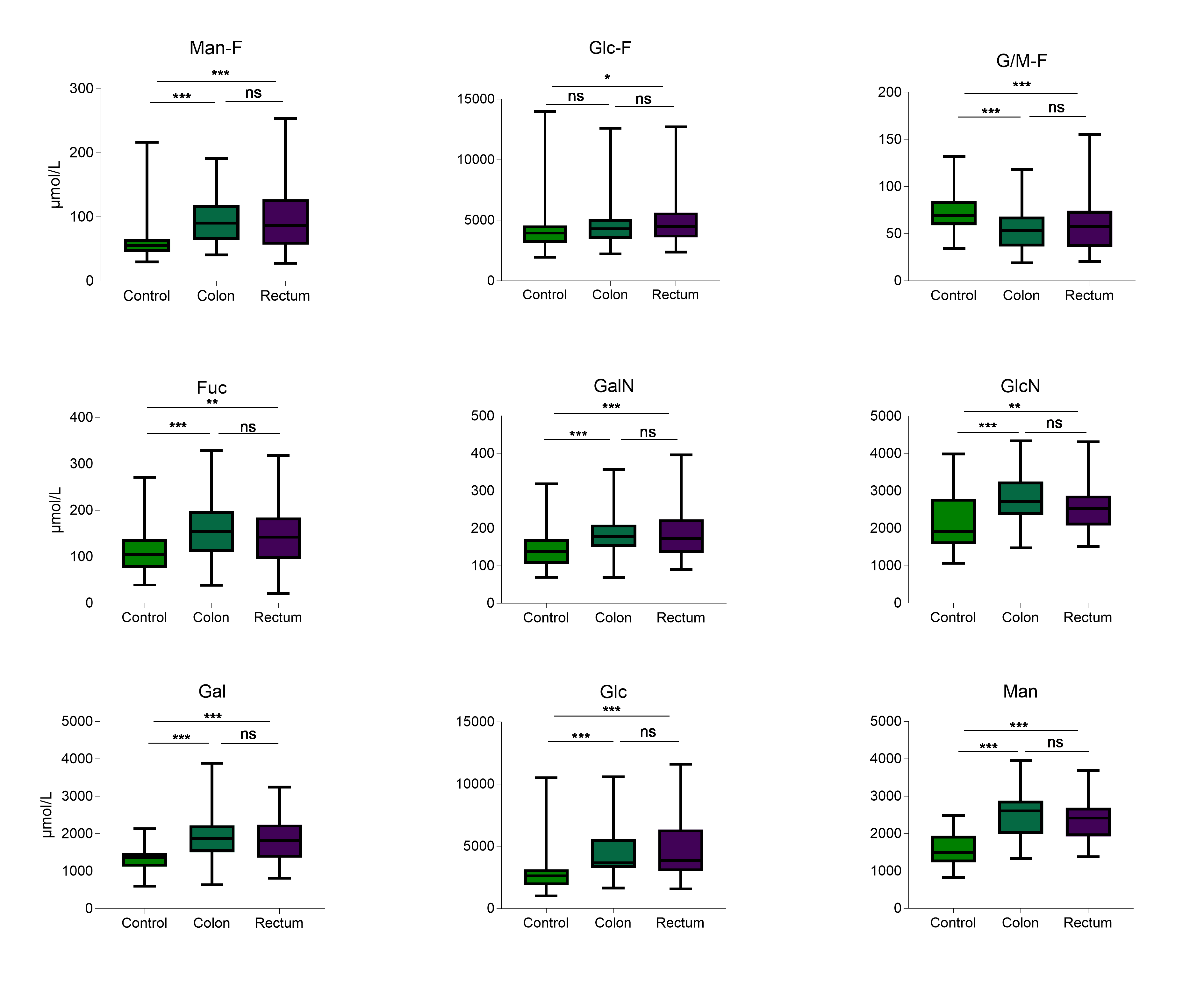

Supplement: Supplementary Figure 1 — Monosaccharide concentrations in sera of colon cancer and rectum cancer had no significant difference. The monosaccharides labeled “- F” are free monosaccharides; Monosaccharides without “- F” labeling are hydrolyzed monosaccharides. ***p ≤ 0. 001, **p ≤ 0.01, *p ≤ 0.05, ns, not significant. [file Image_1.tif]

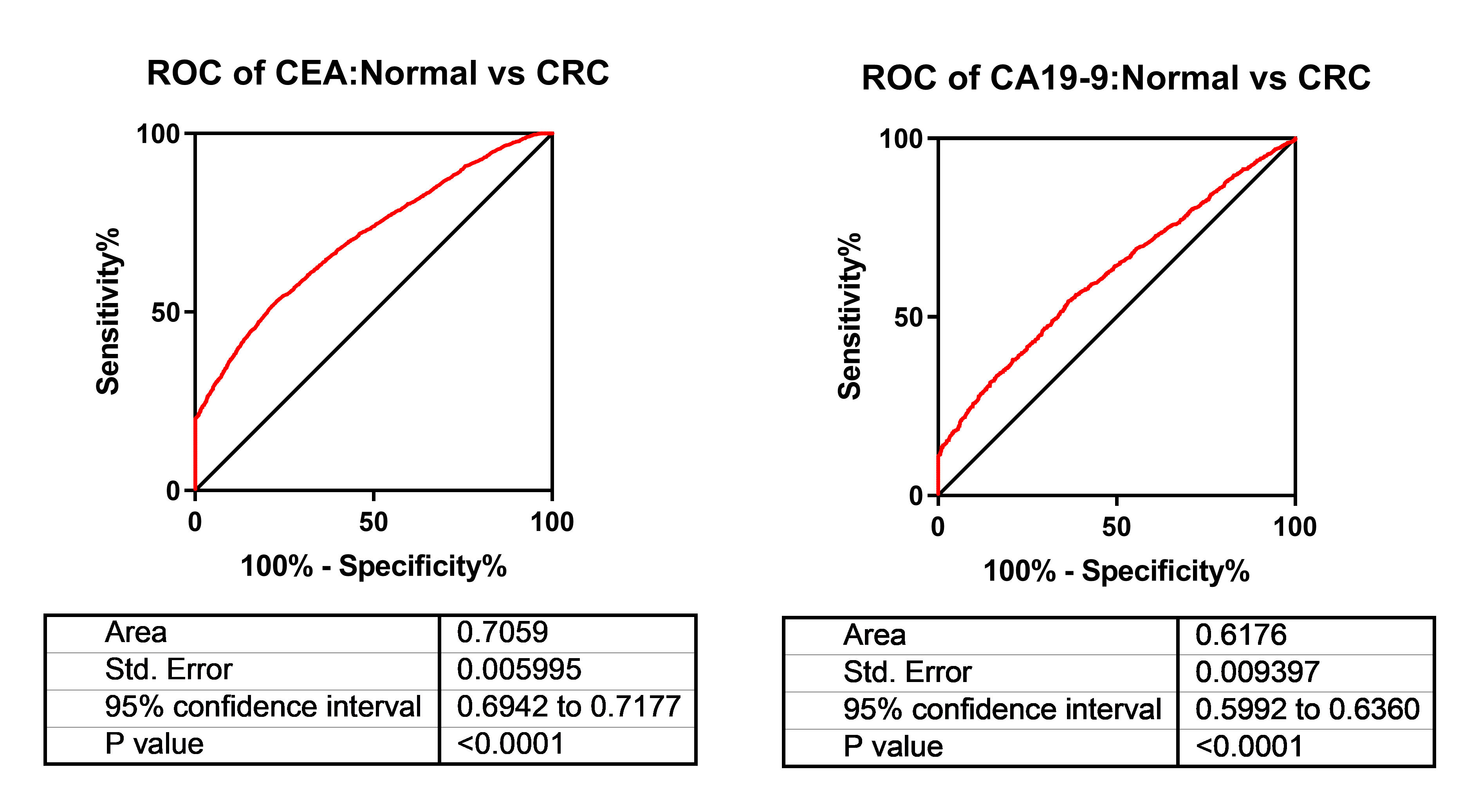

Supplement: Supplementary Figure 2 — ROC curve analysis of CEA (A) and CA199 (B) based on data from January 2013 to January 2018. (A) ROC curve analysis based on clinical CEA data of 2757 healthy individuals and 4513 CRC patients. (B) ROC curve analysis based on clinical CA199 data of 930 healthy individuals and 4166 CRC patients. [file Image_2.tif]
